# Emulsification Properties of Plant and Milk Protein Concentrate Blends

**DOI:** 10.3390/foods14193406

**Published:** 2025-10-01

**Authors:** Mohammadreza Khalesi, Shauna Dowling, Jack Comerford, Ciara Sweeney, Sara Esteghlal, Richard J. FitzGerald

**Affiliations:** 1 Department of Biological Sciences, University of Limerick, V94 T9PX Limerick, Irelandciarasweeney1010@gmail.com (C.S.);; 2 Bernal Institute, University of Limerick, V94 T9PX Limerick, Ireland; 3 Health Research Institute, University of Limerick, V94 T9PX Limerick, Ireland; 4 Department of Food Science and Technology, Shiraz University, Shiraz 71441-65186, Iran; sara.esteghlal@shirazu.ac.ir

**Keywords:** plant protein, milk protein concentrate, blend, emulsion properties

## Abstract

Blending is a promising strategy during the partial replacement of plant with animal proteins. This, however, may lead to alteration in the technofunctional properties of the resultant blends. In this study, partial replacement of milk protein concentrate (MPC) with different plant proteins including soy, rice and pea protein concentrates (SPC, RPC and PPC, respectively) was conducted to determine the effect of blending at different ratios on the technofunctional properties relevant to their emulsification behavior, e.g., emulsion stability, viscosity and water holding capacity (WHC) and oil binding capacity (OBC). It was observed that at equivalent concentrations, the plant protein concentrates had higher apparent viscosities compared to MPC and the blends. RPC–MPC, at all ratios (25:75, 50:50, and 75:25), had a lower OBC when compared with the SPC–MPC and PPC–MPC blends. The lowest OBC was 32.5, for RCP–MPC 25:75, and the highest was 116.0 for SPC–MPC 25:75. The highest solubility of PPC, RPC, and SPC was observed in their blend form at 50:50 (73.2%), 75:25 (86.5%) and 25:75 (71.1%) ratios, respectively. Plant protein–MPC blends showed higher emulsion stability than the individual plant protein concentrates. The highest emulsion stability was 100%, for RPC–MPC 50:50 and 75:25 ratios, PPC–MPC at 50:50 ratio, and SPC–MPC at 25:75 and 100:0 ratios. Among the blends, SPC–MPC 25:75, PPC–MPC 50:50 and RPC–MPC 50:50 showed the most suitable overall emulsification properties. Based on the results, blending MPC with plant protein concentrates led to promising improvements in emulsification behavior relevant to different composite protein ingredient applications.

## 1. Introduction

The global population is progressively increasing, leading to a growing demand for dietary protein [1]. Currently, the proteins used to meet these demands mainly originate from animal sources, e.g., meat and dairy proteins. Milk protein concentrates (MPCs) are dairy ingredients containing 42–85% protein [2]. MPCs are used in products such as in dietary supplements, nutrition bars and sports beverages due to their high level of essential amino acids (EAAs) along with their low lactose content, particularly in the case of higher-protein-content MPCs.

While animal proteins have numerous advantages in food product applications, the latest developments in food sustainability focus on the impact of animal-origin products, particularly in terms of their climate change and economic implications. Accordingly, the demand for alternative protein sources, mainly from plants, is increasing. Many plant proteins have good functional (e.g., emulsification) properties in food applications, which enables them to serve as potential substitutes for animal-origin proteins. Nevertheless, there are some issues that may restrict the widespread application of plant proteins in food products. For instance, some plant proteins contain low levels of certain EAAs and high levels of anti-nutritional factors [3]. In addition, some plant proteins display relatively poor technofunctional properties [4].

Therefore, there is an increased interest in the functionality and nutritional quality of hybrid protein products, i.e., combinations of plant with animal proteins [5,6]. Emerging evidence suggests that blending plant with animal proteins could increase the utilization of plant proteins while improving their overall functionality and nutritional quality. Blending can also be considered a new marketing opportunity for food manufacturers to develop products with novel characteristics (such as improved technofunctionality and sensory properties).

Alves and Tavares [7] stated that the partial replacement of animal protein with plant protein is the first step towards reducing the environmental impacts associated with animal food consumption. There are different studies exploring the emulsification properties of plant protein and animal protein blends. Blending of pea protein isolate (PPI) and whey protein isolate (WPI) improved the functionality of PPI in a mixed-protein system formulation [8]. It has also been shown that blending of skim milk powder with pea protein concentrate (PPC) modified the technofunctional properties of first age infant formula [9]. Blending increased the viscosity and reduced the solubility, but did not change the emulsion stability (ES) of the product. Ho et al. [10] reported that plant-derived emulsifiers generated with soy protein isolate (SPI) and PPI were suitable replacements for dairy proteins including WPI and sodium caseinate. It has been reported that milk protein–soy protein (SP) blends have higher apparent viscosity (η_app_) compared to micellar casein (CN) [11]. For example, potato protein (PP) and whey protein (WP) were blended at different ratios and then used to stabilize oil in water emulsions. The results indicated that by increasing PP content, the viscosity of the emulsions, the amount of adsorbed protein at the oil–water interface, surface protein load, and thickness of the interfacial film were increased, which resulted in emulsions with higher stability. The ratio of 7:3 PP to WP was indicated as the optimal ratio in terms of emulsification aspects [12]. In another study, WPI, SPI, and their blending at 1:1 ratio were used to stabilize emulsions with 5, 10, and 15% oil concentrations. The blended proteins revealed a higher protein adsorption rate than the WPI, and lower than that of SPI. Higher oil concentrations in the emulsions led to an increase in protein adsorption (especially the blend and SPI). The blended proteins resulted in an emulsion with the highest stability [13]. Blending SPI and WPI at different ratios was observed to efficiently decrease the size of emulsion droplets, increase the thickness of the interfacial layer, and enhance emulsion stability. The ratio of 1:9 of SPI to WPI and protein concentration of 0.2 g/kg led to the lowest creaming index and smallest droplet size. The highest protein adsorption to the interfacial layer was observed at a protein concentration of 0.15 g/kg and SPI to WPI mass ratio of 9:1 [14]. Partial replacement of WPI or sodium caseinate with PPI in stabilized emulsions showed that the blend of proteins exhibited higher surface load and droplet size compared to the individual animal proteins. Blending the plant and animal proteins resulted in enhanced storage stability of the emulsions as a result of synergistic effects [15].

Among different plant proteins, soy protein is one of the primary candidates for partially replacing animal proteins. This protein is commercially present in the market and has broad applications owing to its high protein concentration, appropriate cost, and high consumer acceptance among people. Pea protein and rice protein are other promising plant protein sources for the partial replacement of animal proteins as they have desirable properties such as hypoallergenic character with a good balance of amino acids, significant digestibility, high biological value, and anti-cancer and anti-oxidant activities. Rice protein has a nutritional quality similar to or even better than other cereals. In addition to containing 20% proteins with higher lysine and tryptophan content compared to cereal grains, pea protein has 5–20% lower trypsin inhibitors than soybeans. [16,17].

Despite the numerous reports on blending plant and animal proteins, it is still relatively unknown how plant and dairy proteins may behave when in blends. Limited knowledge appears to exist on the impact of blending of dairy and plant proteins on the functionality, e.g., emulsification properties, in different products. Therefore, acquisition of this knowledge may help in the targeted design of balanced blends for different functional and nutritional applications.

The hypothesis is that blending plant with animal proteins has the potential to yield protein mixtures with novel emulsion properties due to the potential for interactive effects between the different origin proteins. This in turn may lead to the development of new functionality and ingredient applications. The objective of this study was to evaluate the impact of blending different plant proteins, i.e., soy protein concentrate (SPC), rice protein concentrate (RPC), and PPC with MPC at different ratios on properties relevant to the emulsification behavior of blends. In addition, the optimal plant proteins and blending ratios in terms of improved emulsification properties will be introduced. To our knowledge, this appears to be the first report on the emulsion properties of plant protein–MPC blends.

## 2. Materials and Methods

### 2.1. Materials

SPC, PPC and RPC from Pulsin Ltd. (Gloucester, UK) were obtained at a local health food store and MPC85 (85% (*w*/*w*) protein) was obtained from a commercial manufacturer. Corn oil was purchased from a local food market. Sodium hydroxide (NaOH) and acetic acid were from Fisher Scientific (Dublin, Ireland). Kjeldahl catalyst tablets, sulfuric acid (>98%), boric acid, 2-mercaptoethanol, methanol, protein molecular mass markers (6.5–200.0 kDa) and Sudan III were from Sigma-Aldrich (Dublin, Ireland). Hexane was from Honeywell International Inc. (Dublin, Ireland). Coomassie R, Laemmli buffer, Mini-Protean TGX 4–20% pre-cast polyacrylamide gels were from Bio-Rad Laboratories Inc. (Hercules, CA, USA) and sodium dodecyl sulfate (SDS) was from National Diagnostics (Atlanta, GA, USA).

### 2.2. Proximate Analysis and pH Determination

Moisture, ash, lipid and protein contents were determined (*n* = 3) according to Khalesi and FitzGerald [18].

### 2.3. Blending of Plant Protein Samples with MPC

**Figure 1:**
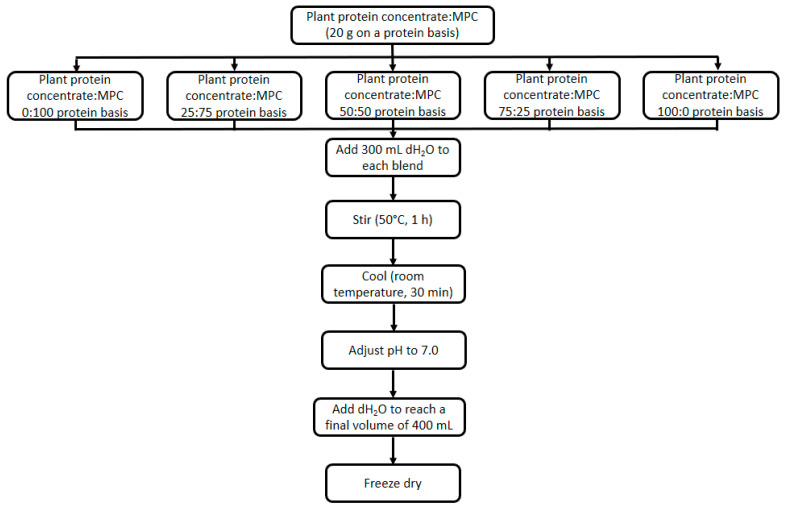
Schematic of the process of preparing blends of the plant protein concentrates (i.e., soy (SPC), pea (PPC) and rice (RPC) protein concentrates) and milk protein concentrate (MPC).

Different blends having different ratios of proteins from plant sources and MPC85 were generated as schematically outlined in Figure 1. In addition, the quantities of each protein at each ratio are shown in Table 1.

### 2.4. Technofunctional Property Analysis

#### 2.4.1. Emulsification

Freeze-dried samples of each individual plant protein concentrate, MPC and the plant protein–MPC blends were resuspended with dH_2_O and adjusted to pH 7.0 to give a 0.025% (*w*/*v*) protein suspension. Sudan Red III (40 mg) was added to 1 L of corn oil, after which 6 g was added to 14 g of each protein sample suspension. Samples were then homogenized using an Ultra-Turrax (IKA T25, Staufen, Germany) for 1 min at 16,000 rpm in order to create an emulsion. Immediately after homogenization, an aliquot of sample (18 µL) was 100 fold diluted with 0.1% (*w*/*v*) SDS to reach the volume of 1.8 mL. The absorbance (A, λ500) of the bottom half of the emulsion sample was measured (*n* = 3) using a UV–Vis 1800 spectrophotometer (Shimadzu, Canby, OR, USA) at T0 and 30 min (T30) after emulsion formation. ES was determined according to Equation (1):

(1) $$ES(\%)=\frac{A_{T30}}{A_{T0}}\times 100$$

where AT30 and AT0 represent the absorbance (λ500) at T30 and T0 (min), respectively.

#### 2.4.2. Apparent Viscosity (η_app_)

An aliquot (16 mL) of each suspension equivalent to 5% (*w*/*v*) protein prepared after reconstitution of the freeze-dried samples was analyzed using a Brookfield DV-II viscometer (Analytica, Dublin, Ireland) at 30 °C (*n* = 3) at a shear rate of 6 s^−1^ for the PPC, SPC and their MPC blends and at 100 s^−1^ for the RPC-containing samples. The η_app_ of MPC was measured at both share rates (i.e., 6 and 100 s^−1^).

#### 2.4.3. Water Holding Capacity (WHC) and Oil Binding Capacity (OBC)

The WBC/OBC for the different freeze-dried blends was determined (*n* = 3) by resuspension of each sample/blend in dH_2_O/corn oil to reach a final concentration of 5% (*w*/*v*) on a protein basis, vortexed for 30 s followed by centrifugation (320R Hettich centrifuge, Tuttlingen, Germany) at 3000 g at 20 °C for 30 min and then, the supernatant (water/oil) was removed. The WHC (g dH_2_O/g protein)/OBC (g oil/g protein) was calculated according to Equation (2) [18].

(2) $$WHC/OBC(\%)=\frac{W3-W1}{W2}\times 100$$

where W1, W_2_ and W_3_ are the mass of the freeze-dried sample, weight of protein in each powder sample (as determined in Section 2.2), and the weight of residue after centrifugation followed by removal of the supernatant, respectively.

#### 2.4.4. Solubility

To determine the solubility of the proteins/blends (*n* = 3), certain amount of each sample was dispersed in dH_2_O to reach 5% (*w*/*v*, *protein basis*) concentration and then stirred for 30 min at RT to ensure complete hydration. The dispersion was then centrifuged (320R Hettich centrifuge, Tuttlingen, Germany) at 1000 g for 10 min at 25 °C. The supernatant was carefully collected and its total solids content was determined after drying at 95 °C for 6 h in a vacuum oven (OVA03100, Gallenkamp Ltd., Loughborough, UK). Solubility of the samples was then calculated based on the below eq [18].

(3) $$Solubility(\%)=\frac{Total\;solids\;of\;supernatant}{Total\;solids\;of\;protein\;dispersion}\times 100$$

### 2.5. Statistical Analysis

Each test was conducted three times (*n* = 3). Data values were presented as the mean ± standard deviation (SD). One-way analysis of variance (ANOVA) followed by the Tukey post hoc comparison test was carried out to test for significant differences using Minitab^®^ Release 15 for Windows. A *p* value < 0.05 was considered statistically significant.

## 3. Results and Discussion

### 3.1. Proximate Analysis and Sample Properties

As shown in Table 2, it is evident that there was some variation in the moisture, protein, ash and the lipid contents of the different test samples. The moisture content in the samples ranged from 1.64 to 5.58% with the lowest value being for RPC and the highest value for SPC. Similarly to our results, Kaspchak et al. reported that among soy, pea, and rice protein isolates, the lowest and highest moisture content were for rice protein isolate (1.43%) and soy protein isolate (3.04%), respectively [19]. MPC had the highest overall protein content (84.17 ± 0.79%), while among the plant protein concentrates, SPC had the highest protein content (81.11 ± 0.77%). The protein content obtained for PPC was 71.01 ± 0.25%. Elsewhere, the chemical analysis of pea, soy, and rice proteins indicated that the protein content was in the range of 76.96–86.36%, with the highest protein content for soybean protein [17]. Similarly, proximate analysis of the same proteins in another study indicated the amount of 89.99%, 88.00%, and 82.76% protein for soy, pea, and rice protein isolates, respectively [19]. The lipid content of MPC85 (1.31 ± 0.07%) was the lowest among the samples as it is manufactured from skim milk. Significant differences were found between the lipid content in each of the plant protein samples. The lipid content in RPC (9.70 ± 0.37%) and PPC (8.13 ± 0.17%) was higher than in SPC (1.79 ± 0.11%). The ash content showed less variance, the highest mean value being 6.96% in MPC and the lowest being 5.47% for SPC. PPC had the highest mean ash level (6.48%) among the plant protein samples. The amount of measured ash by Zhao et al. in soy, rice, and pea protein samples was reported to have lower values, 0.24, 3.83, and 4.01 for rice, pea, and soy proteins, respectively [17]. The lower ash content may be attributed to different varieties of the plants and the protein extraction method.

The reconstitution pH values recorded ranged from pH 6 to 8, with RPC being slightly acidic (pH 6.09) and PPC being slightly basic (pH 8.00). The mean pH of SPC was similar to MPC (7.15 vs. 7.09).

### 3.2. Technofunctional Properties

#### 3.2.1. Apparent Viscosity (η_app_)

**Figure 2:**
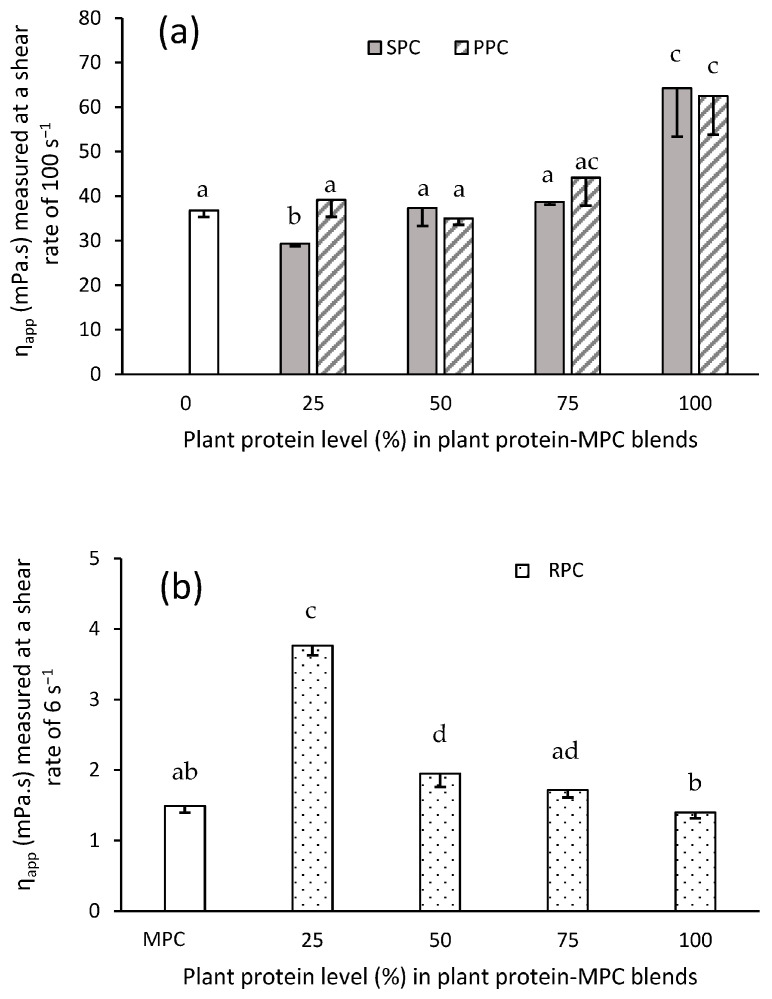
The apparent viscosity (η_app_) (**a**) of soy (SPC) and pea (PPC) protein concentrates blended with milk protein concentrate (MPC) at different ratios, i.e., 0:100, 25:75, 50:50, 75:25 and 100:0, at a shear rate of 6 s^−1^, and (**b**) of rice protein concentrate (RPC) blended with MPC at different ratios, i.e., 0:100, 25:75, 50:50, 75:25 and 100:0, at a shear rate of 100 s^−1^. Values represent the mean ± SD (*n* = 3). Different letters represent significant differences (*p* < 0.05).

The η_app_ varied across the plant blends (Figure 2). Overall, SPC and PPC produced the most viscous suspensions. The η_app_ for SPC (64.3 ± 10.9 mPa.s) and PPC (62.5 ± 8.7 mPa.s) were not significantly different (*p* > 0.05). Both 100% SPC and PPC gave a higher viscosity value than MPC (36.8 ± 1.2 mPa.s) at a similar shear rate. Previously, the η_app_ of SPC was reported to be similar to sodium caseinate but was two times higher when compared to WPI [20]. The results herein demonstrated that PPC and SPC have, under certain circumstances, elevated viscosity properties which may be desirable for emulsions as well as in the formulation of high viscosity requiring products, e.g., plant-based yogurts and ice cream. The η_app_ of RPC (1.4 ± 0.1 mPa.s) was not significantly (*p* > 0.05) different from MPC (1.5 ± 0.1 mPa.s) when tested at a similar shear rate (i.e., 6 s^−1^). The mean viscosity values of the SPC–MPC blends tended to increase as the proportion of SPC in the blends increased. This is associated with the higher η_app_ of SPC compared to MPC. The η_app_ associated with the blends generated with PPC was higher (*p* < 0.05) than those generated with SPC, except when at a ratio of PPC–MPC of 50:50 (Figure 2a). A previous study showed that the inclusion of PPC in infant formula produced with skim milk (50:50) enhanced the viscosity while the inclusion of faba bean protein at the same ratio did not change the overall viscosity [21]. The η_app_ of WPI was reported to be significantly increased on blending with PPI [22]. The η_app_ of sodium caseinate was reported to be higher than PPI and a PPI-sodium caseinate hybrid blend [23]. The reconstituted suspensions of the RPC–MPC blends had the lowest η_app_ among the plant–MPC blends (Figure 2b). Increasing the quantity of RPC did not increase the η_app_, with the RPC–MPC 25:75 blend yielding the highest η_app_ value (3.76 ± 0.13 mPa.s) among the RPC–MPC blends (*p* < 0.05). The results showed that the hybrid blends created with RPC and MPC had a higher η_app_ compared to MPC and RPC alone, indicating possible interactions between the RPC and MPC protein suspensions.

Overall, the PPC–MPC and SPC–MPC blends may prove useful for high viscosity requiring applications. It should also be noted that the presence of other non-proteinaceous components may cause differences between the η_app_ of various plant protein ingredients. In addition, most of the blend samples gave η_app_ values higher than that for MPC alone, suggesting the possibility of replacement of MPC with plant proteins for high viscosity requiring purposes. In general, higher viscosity is associated with better emulsification properties as in a more viscose medium, the mobility of the oil droplets is reduced, which results in lower coalescence within the emulsion [24].

#### 3.2.2. WHC and OBC

**Figure 3:**
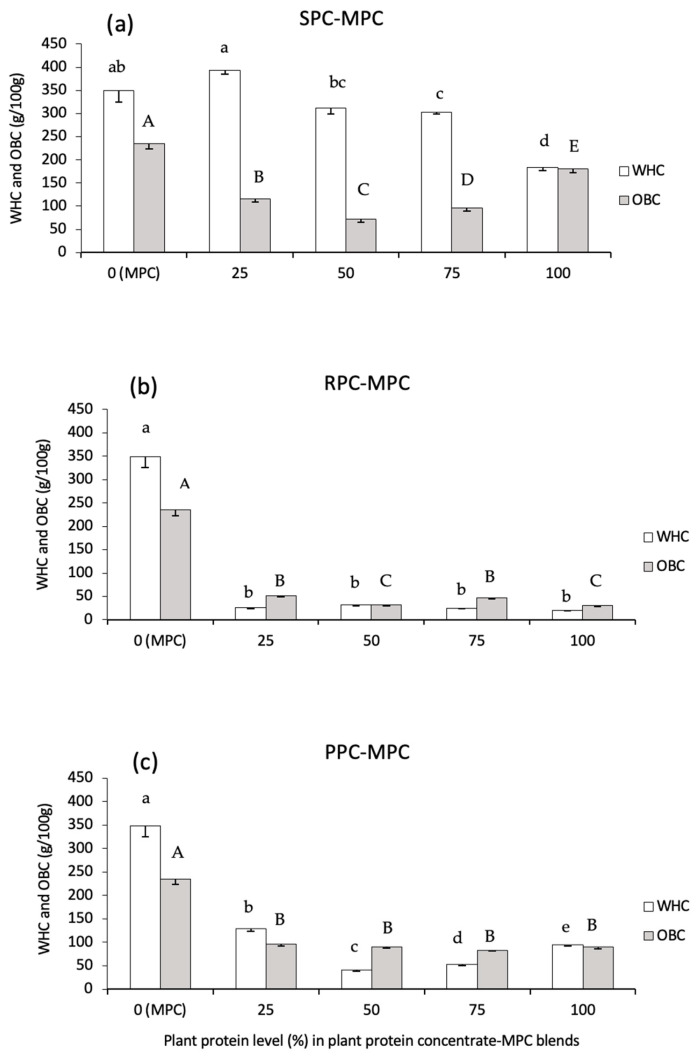
Water holding (WHC) and oil binding capacity (OBC) of (**a**) soy (SPC), (**b**) rice (RPC) and (**c**) pea protein concentrates (PPCs) blended with milk protein concentrate (MPC) at different ratios, i.e., 0:100, 25:75, 50:50, 75:25 and 100:0. Values represent the mean ± SD (*n* = 3). Different lower case letters represent significant differences (*p* < 0.05) for WHC values, and different upper case letters represent significant differences (*p* < 0.05) for OBC values.

MPC had a higher (*p* < 0.05) WHC (349 ± 24 g water/100 g protein) compared to the individual plant protein concentrates tested (Figure 3). The extensive interaction between protein components, especially the CNs, in MPC and water molecules is considered the main reason for the high WHC of MPC. In addition, the plant protein concentrates had a higher lipid content, thus expectedly; they showed a lower affinity to retain water compared to MPC. SPC had a higher (*p* < 0.05) WHC (184 ± 8 g water/100 g protein) than RPC (26 ± 2 g water/100 g protein) and PPC (129 ± 5 g water/100 g protein). Some specific protein components of SPC (particularly the 11S globulin) have previously been shown to contribute to the WHC and to the formation of stable protein gels [25]. The higher WHC of SPC compared to PPC and RPC may also be associated with the lower lipid content of the SPC ingredient studied herein. Among the blended samples, SPC–MPC 25:75 (394 ± 9 g water/100 g protein) had the highest (*p* < 0.05) WHC. As the proportion of SPC increased and MPC decreased in the SPC–MPC blends, the WHC decreased. All SPC–MPC blends had a notably higher WHC in comparison to the RPC–MPC and PPC–MPC blends. Minimal variation was observed in the WHC between any of the RPC–MPC blends where the RPC–MPC blends had the lowest WHC (23–33 g water/100 g protein). The WHC of the PPC–MPC blends was significantly lower (*p* < 0.05) than the MPC sample. The WHC of the PPC–MPC 25:75 blend (129 ± 5 g water/100 g protein) was higher than 100% PPC (94 ± 2 g water/100 g protein) (*p* < 0.05). These results indicate interactions between SPC–MPC (25:75) resulting in an improvement in the WHC. This may be beneficial for the partial replacement of MPC for applications where high WHC and gelation properties are required, e.g., in yogurt and cheese-type products.

Variability in the results of OBC of individual plant proteins was observed which may be associated with the composition of the plant proteins, especially the lipid and protein contents and also the surface located composition of the powder particles. As shown in Figure 3, no plant protein concentrates or plant protein–MPC blend reached an OBC similar to that of MPC (239 g oil/g protein). This may be related to the presence of high levels of surface lipid in MPC85 which has been shown to consist of >15% of its surface composition [26]. In addition, the low OBC of plant proteins has previously been reported to be due to a large proportion of hydrophilic protein groups on their surfaces [27]. The OBC of SPC (180 ± 8 g oil/100 g protein) and SPC–MPC blends (ranging between 66 and 123 g oil/100 g protein) was highest amongst the plant protein concentrates and plant protein–MPC blends. RPC and RPC–MPC blends performed the poorest across all plant protein concentrates and plant protein–MPC blends in terms of OBC. Among the RPC–MPC blends, RPC–MPC 25:75 yielded the highest OBC at 51 ± 2 g oil/100 g protein, while the lowest OBC (33 ± 2 g oil/100 g protein) was seen for RPC–MPC 50:50. The OBC of PPC was 89 ± 3 g oil/100 g protein, which is in the range of previous reports showing that the OBC of commercial PPC was ~100 g oil/100 g protein. The ratio of 11S to 7S globulins was suggested to have an impact on the OBC of PPI [28]. Among PPC–MPC blends, PPC–MPC 25:75 yielded the highest OBC at 96 ± 4 g oil/100 g protein, while the lowest OBC (82 ± 1 g oil/100 g protein) was seen for PPC–MPC 75:25. These results showed that the blending of plant with milk proteins did not increase the OBC of the samples. The higher OBCs in the SPC and PPC blends (in comparison with the RPC blends) may be an advantage for some functionalities such as for emulsification and applications related to the formulation of breads, cakes and muffins.

#### 3.2.3. Solubility (%)

**Figure 4:**
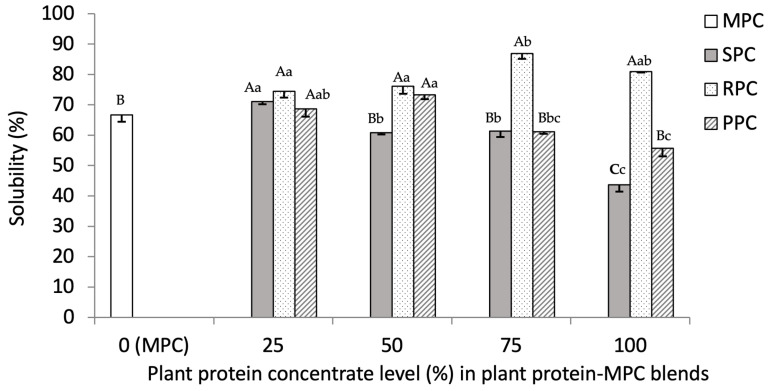
Solubility (%) of soy (SPC), pea (PPC) and rice protein concentrates (RPC) blended with milk protein concentrate (MPC) at different ratios, i.e., 75:25, 50:50 and 25:75. Values represent the mean ± SD (*n* = 3). Different small letters indicate significant differences among different ratios of a constant sample and different capital letters indicate significant differences among different samples at a constant ratio (*p* value < 0.05).

As shown in Figure 4, the lowest solubility of the plant protein concentrates tested was associated with SPC (43.70 ± 2.33%) and PPC (55.74 ± 2.75%), while the highest was associated with RPC (80.94 ± 0.35%). The solubility varied between the different blends within each plant protein sample highlighting the differences in their interactions with MPC (Figure 4). The mean solubility of the RPC–MPC blends was higher than both the SPC– and PPC–MPC blends. Overall, the RPC–MPC 75:25 blend gave the highest solubility (86.89 ± 1.70%) among the blends (*p* < 0.05). The solubility of PPC was lower than RPC (*p* < 0.05). However, blending PPC with MPC increased its solubility, with the highest being associated with PPC–MPC 50:50 and 25:75. A higher proportion of PPC, however, reduced solubility. SPC had the poorest solubility among the individual plant proteins. Blending SPC–MPC enhanced the overall solubility of SPC. Among the SPC–MPC blends, the highest solubilization was for the SPC–MPC 25:75 blend. The enhanced solubility of some plant protein–MPC blends was evidence for a synergistic relationship between plant proteins and MPC and their interactions with the aqueous phase.

**Figure 5:**
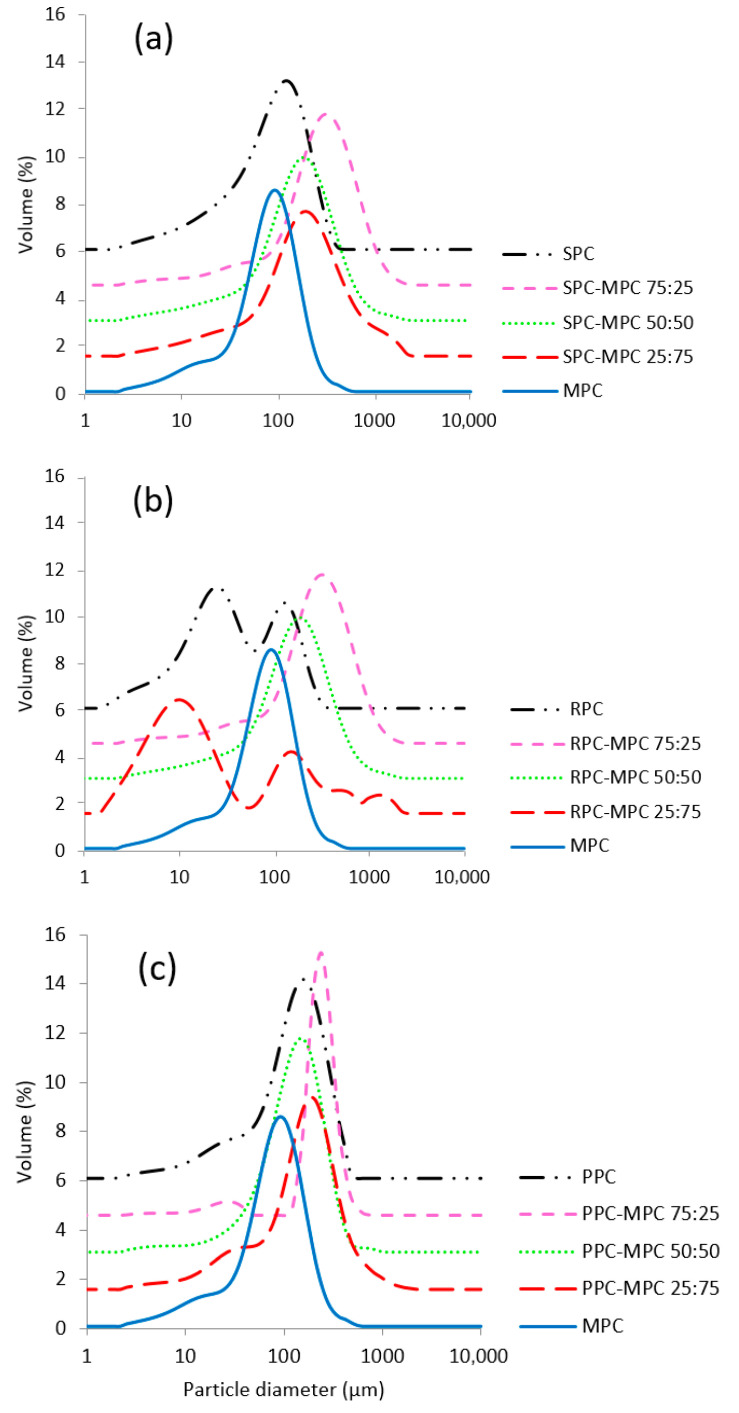
Particle size distribution profiles of (**a**) pea (PPC), (**b**) rice (RPC) and (**c**) soy protein concentrates (SPC) blended at different ratios with milk protein concentrate (MPC) at different ratios, i.e., 75:25, 50:50 and 25:75 (*n* = 3).

In addition, the particle size distributions of 5% (*w*/*v*, protein) of the aqueous powder suspensions of each plant protein concentrate and the blends were measured (Section 2.4.4) using laser light scattering (Table 3). The mean Sauter diameter D [3,2 of MPC was 38.55 ± 2.11 µm, which was lower than for SPC and PPC, but was larger than for the RPC sample. The D[3,2] for all tested samples ranged between 8.01 and 101.79 µm, with the RPC–MPC blends having the lowest D[3,2] on average (ranging between 8.01 and 23.33 µm). The low D[3,2] values for the RPC–MPC blends were in line with their higher solubility and lower η_app_ in comparison to PPC– and SPC–MPC blends. However, the polymodal particle size distribution seen in the RPC samples (Figure 5) suggests that these suspensions do not remain stable over time, as those particles may further coalesce. This polydispersity was not observed in either the PPC or the SPC samples. The D[3,2] associated with the SPC–MPC and PPC–MPC blends was in the range of 57.52–101.79 µm. Among the SPC blends, the SPC–MPC 75:25 sample had the highest D[3,2] (88.36 ± 1.93 µm). Similarly, among the PPC blends, the PPC–MPC 75:25 blend had the highest D[3,2] (98.50 ± 3.29 µm). These results showed that the presence of a lower proportion of MPC in SPC–MPC and PPC–MPC blends increased the PS, which is in accordance with the lower solubility observed for these blends.

The SSA of the blends was compared (Table 3). The PPC–MPC and SPC–MPC blends gave the lowest SSA. As expected, the RPC–MPC 25:75 blend, which had the lowest D [3,2], presented the highest SSA (0.64 m2/g). In general, particles with smaller sizes and larger surface areas have positive implications for the stability of emulsified foods [29].

According to these results and previous literature, the formation of soluble plant protein–MPC blends depends on the plant protein source, the proportion of the plant protein in the blend and it also depends on the interactions between the plant proteins and CN/WP fractions in MPC.

#### 3.2.4. Emulsion Stability (ES%)

Among samples with 100% plant protein, the lowest ES was for the PPC emulsion (51%) and the highest was for SPC (100%). In addition, the ES of MPC was 74%, lower than SPC and RPC and higher than PPC. The emulsification capacity of proteins depends on various factors including their extraction conditions, molecular weight, size, structural rigidity and conformation, and surface charge [30]. The combined effect of these parameters determine the emulsion stability and the impact of one or several parameters may be dominant in certain cases. Based on the results of particle size and solubility measurements (Section 3.2.2), SPC exhibited the smallest particle size after RPC, which results in its faster adsorption to the oil/water interface and therefore, higher emulsification capacity. In addition a high protein content of this sample, it had the highest apparent viscosity, which could slow down droplet movements in the continuous phase and enhance the emulsion stability. The lowest ES of PPC may be attributed to its considerably larger size compared to other samples as well as its lower protein content compared to other proteins (Table 3). Different mechanisms may also be involved in stabilization of emulsions by different proteins. For instance, investigating the mechanisms of emulsion stabilization of rice protein, pea protein and potato protein in a research study indicated that rice protein forms a physical barrier around oil droplets while pea protein and potato protein from a film layer around the droplets [31].

**Figure 6:**
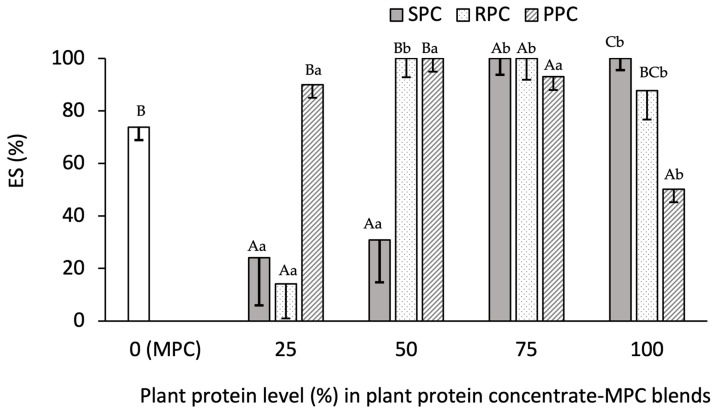
Emulsion stability (ES%) of soy (SPC), rice (RPC) and pea protein concentrates (PPC) blended at different ratios, i.e., 0:100, 25:75, 50:50, 75:25 and 100:0, with milk protein concentrate (MPC) (*n* = 3). Different small letters indicate significant differences among different ratios of a constant sample and different capital letters indicate significant differences among different samples at a constant ratio (*p* value < 0.05).

There were clear differences in the impact of various proportions of plant proteins on ES. Blends which yielded 100% stability after 30 min holding at room temperature were RPC–MPC 50:50, RPC–MPC 75:25, SPC–MPC 75:25, SPC–MPC 100:0 and PPC–MPC 50:50 (Figure 6). The RPC–MPC 25:75 (11%), SPC–MPC 25:75 (21%) and SPC–MPC 50:50 (31%) yielded the lowest ES. Incorporation of 75% plant protein into MPC yielded a high ES in all cases. Incorporation of 50% plant protein to MPC also yielded a high ES in the case of the RPC– and PPC–MPC blends.

The results of ES suggest PPC had the highest extent of interaction with the milk proteins and perhaps the PPC–MPC blends had the highest interfacial energy among the blends, given that the 100% PPC sample exhibited the lowest ES. This considerably improved with the introduction of MPC and was highly stable at a ratio of 50:50 (100%). Furthermore, the PPC–MPC 25:75 emulsion showed higher ES (90%) compared to the other two plant protein samples at the same ratio. This implies a strong interfacial film being generated by the PPC–MPC blend, which was in accordance with a previous report on the emulsion stability of WPI-PPI blends (50:50) [10]. A synergistic action between pea and milk protein has also been highlighted by Hinderink et al., [32] who observed PPI-sodium caseinate and PPI-WP emulsions which remained stable over 14 d, unlike emulsions formed by either PPI and sodium caseinate alone. The rate of adsorption of the blend of dairy proteins (WPI and sodium caseinate) and PPI at the air-water interface was higher than individual proteins showing a synergistic effect arising from blending. In addition, blends of sodium caseinate and PPI had an improved interfacial strength (which is an indication for ES) compared to sodium caseinate alone and thicker films were formed compared to all individual proteins. The emulsion activity of the blends generated with PPI and WPI was recently shown to be higher than the PPI alone [8]. This effect may be associated with reduced flocculation and coalescence by the proteins due to electrostatic interaction between surface protein charges. In addition, Kristensen et al., [8] found that pea proteins are capable of adsorbing to the oil–water interface after introduction to a pre-adsorbed WP interface. On the other hand, Hinderink et al. [33] found that a pre-adsorbed PPC at the oil–water interface can be replaced with β-lactoglobulin. The addition of PPC to infant formula has been reported to have no effect on the emulsion characteristics of the product [34].

It was also found herein that the lower proportion of SPC was less beneficial in the SPC–MPC (25:75) blend system for ES. Increasing the proportion of SPC improved ES. A major increase in ES was observed by increasing the SPC content in the SPC–MPC blend from 50 to 75%. This may be indicative of the high ES of SPC per se, which was previously reported by Molina et al. [35]. Ji et al. [36] reported higher long-term ES in sodium caseinate-SPC emulsions compared to sodium caseinate or SPI stabilized emulsions. Synergic effects on the interfacial strength and viscoelastic film at the air/water interface were also reported for β-conglycinin (7S) and β-lactoglobulin (50:50) compared to the individual proteins [37].

The long-term stability of the prepared emulsions was not evaluated in this work. However, due to the potential application of the emulsions stabilized by plant and animal protein blends in various food products, analysis of long-term stability of emulsification properties under different storage conditions appears to be required.

## 4. Conclusions

Blending plant proteins with animal proteins is an efficient method to fulfill the increasing global demand for health promoting foods and to decrease the impact of humans on the environment. In addition, the demonstration of new functions for novel protein ingredients compared to existing highly consumed animal-origin proteins is necessary to expand the protein market. Incorporation of plant proteins (SPC, PPC and RPC) with MPC in different ratios in this study showed that these blends may be successfully used for partial replacement of MPC. Multiple blends arising from SPC–, RPC– and PPC–MPC were shown to have functional properties that may be useful in specific food applications. Among the blends, SPC–MPC 25:75, PPC–MPC 50:50 and RPC–MPC 50:50 were shown to be the most suitable in regard to their overall emulsification properties. These findings showed that while various plant protein sources had different emulsion properties, the interactions of these proteins with MPC at certain ratios enhanced their ES. This is advantageous for the partial replacement of MPCs with plant proteins to yield highly stable hybrid emulsions for different applications such as in the manufacture of soups and sauces. Different types of protein–protein interactions take place depending on the characteristics of the individual proteins in the plant protein concentrates and in MPC. Therefore, there is a need for further studies to unravel the nature of these interactions and the potential impact of conventional/novel processing conditions on same. Considering the important impact of solubility on emulsification capability of proteins and the relative low solubility of plant proteins, further investigations should be carried out to introduce the optimal concentration range of plant proteins, resulting in the highest solubility. The long-term stability of the emulsions under various conditions should also be investigated for their application in food industry.

## Figures and Tables

**Table 1:** Quantities of each protein type at each ratio.

Protein Type	Plant Protein to Milk Protein Ratio				
	100:0	75:25	50:50	25:75	0:100
Plant protein (g)	20	15	10	5	0
Milk protein (g)	0	5	10	15	20
Total weight of blend (g)	20	20	20	20	20

**Table 2:** Moisture, protein, ash and lipid content along with reconstitution pH (at 0.05% (*w*/*v*) on a protein basis) of soy (SPC), rice (RPC) and pea protein concentrates (PPC) and milk protein concentrate (MPC).

Sample	Moisture	Protein	Ash	Lipid	pH
	(%)				
SPC	5.58 ± 1.57 ^a^	81.11 ± 0.77 ^a^	5.47 ± 0.27 ^a^	1.79 ± 0.11 ^a^	7.15 ± 0.06 ^a^
RPC	1.64 ± 0.41 ^b^	80.04 ± 0.88 ^a^	5.50 ± 0.29 ^a^	9.70 ± 0.37 ^b^	6.09 ± 0.04 ^b^
PPC	3.86 ± 0.01 ^a^	71.01 ± 0.25 ^b^	6.48 ± 0.47 ^b^	8.13 ± 0.17 ^c^	8.00 ± 0.06 ^c^
MPC	4.82 ± 0.02 ^a^	84.17 ± 0.79 ^c^	6.96 ± 0.16 ^b^	1.31 ± 0.07 ^d^	7.09 ± 0.03 ^a^

Values represent the mean ± SD (*n* = 3). Different letters represent significant differences within each column (*p* < 0.05).

**Table 3:** Mean Sauter diameter D[3,2] and specific surface area (SSA) values for soy (SPC), rice (RPC) and pea (PPC) protein concentrates, respectively, blended at 75:25, 50:50 and 25:75 with milk protein concentrate (MPC).

	D[3,2] (µm)		
Plant protein–MPC	SPC–MPC	RPC–MPC	PPC–MPC
0:100	38.55 ± 2.11 ^a^	38.55 ± 2.11 ^a^	38.55 ± 2.11 ^a^
25:75	59.47 ± 1.67 ^b^	9.42 ± 1.41 ^d^	58.60 ± 1.91 ^b^
50:50	59.37 ± 1.85 ^b^	22.07 ± 1.26 ^e^	61.35 ± 2.20 ^b^
75:25	88.36 ± 1.93 ^c^	12.18 ± 1.44 ^d^	98.50 ± 3.29 ^g^
100:0	34.82 ± 1.90 ^a^	17.04 ± 1.60 ^f^	47.89 ± 2.03 ^h^
	**SSA (m^2^/g)**		
0:100	0.16 ± 0.02 ^AC^	0.16 ± 0.02 ^AC^	0.16 ± 0.02 ^AC^
25:75	0.10 ± 0.02 ^BC^	0.64 ± 0.01 ^D^	0.10 ± 0.02 ^BC^
50:50	0.10 ± 0.02 ^BC^	0.27 ± 0.01 ^E^	0.10 ± 0.02 ^BC^
75:25	0.07 ± 0.02 ^B^	0.49 ± 0.01 ^F^	0.06 ± 0.03 ^B^
100:0	0.17 ± 0.02 ^A^	0.35 ± 0.02 ^G^	0.13 ± 0.02 ^C^

Values represent the mean ± SD (*n* = 3). Different lower-case letters represent significant differences between D[3,2] values and different upper-case letters represent significant differences between SSA (*p* < 0.05) values.

## Data Availability

The data presented in this study are available on request from the corresponding author.

## Abbreviations

The following abbreviations are used in this manuscript:

AA	amino acid
η_app_	apparent viscosity
CN	casein
DF	diafiltration
ES	emulsion stability
EAA	essential amino acids
MPC	milk protein concentrate
MPI	milk protein isolate
MW	molecular weight
OBC	oil binding capacity
PS	particle size
PP	pea protein
PPC	pea protein concentrate
PPI	pea protein isolate
PAGE	polyacrylamide gel electrophoresis
RP	rice protein
RPC	rice protein concentrate
RPI	rice protein isolate
SDS	sodium dodecyl sulfate
SP	soy protein
SPC	soy protein concentrate
SPI	soy protein isolate
SSA	specific surface area
TS	total solids
UF	ultrafiltration
WHC	water holding capacity
WP	whey protein
WPC	whey protein concentrate
WPH	whey protein hydrolysate
WPI	whey protein isolate
